# Efficacy and safety of a novel technique using endoscopic ultrasound—guided fine-needle injection of botulinum toxin and balloon dilation in achalasia

**DOI:** 10.1016/j.igie.2025.10.014

**Published:** 2025-10-18

**Authors:** Aqsa Khan, Harishankar Gopakumar, Ricardo J. Marrero Torres, Ahmad Abulawi, Talia Malik, Dushyant Singh Dahiya, Mariajose Rojas DeLeon, Amelia Roebuck, Neil R. Sharma

**Affiliations:** 1 Department of Internal Medicine, Parkview Health, Fort Wayne, Indiana, USA; 2 Department of Gastroenterology and Hepatology, University of Illinois College of Medicine at Peoria, Peoria, Illinois, USA; 3 Interventional Oncology and Surgical Endoscopy (IOSE), Parkview Health, Fort Wayne, Indiana, USA; 4 Department of Gastroenterology and Hepatology, Albany Medical Center, Albany, New York, USA; 5 Department of Gastroenterology, Medical College of Wisconsin, Milwaukee, Wisconsin, USA; 6 Department of Gastroenterology and Hepatology, The University of Kansas School of Medicine, Kansas City, Kansas, USA; 7 Health Services and Informatics Research Department, Parkview Mirro Center for Research and Innovation, Parkview Health, Fort Wayne, Indiana, USA; 8 Department of Biostatistics & Health Data Science, Indiana University School of Medicine Fort Wayne, Fort Wayne, Indiana, USA

## Abstract

**Background and Aims:**

Achalasia is an esophageal motility disorder characterized by the inability of the lower esophageal sphincter to relax. Endoscopic methods are currently the mainstay treatment of achalasia and include local administration of botulinum toxin (Botox; Allergan Aesthetics, Westport, Ireland), pneumatic dilation, and peroral endoscopic myotomy. Botox injections traditionally have been performed by standard endoscopy, which lacks precision requiring frequent interventions. Our study introduces a novel technique using endoscopic ultrasound—guided fine-needle injection of Botox combined with controlled radial expansion balloon dilation (EUS-BTID) for treating achalasia. We hypothesize that this combined approach will enhance the efficacy and offer a favorable safety profile compared with standard endoscopic Botox injection.

**Methods:**

We conducted a single-center retrospective study to evaluate the efficacy and safety of a novel technique using EUS-BTID to treat achalasia using electronic medical records.

**Results:**

Our study included a total of 43 patients, with a mean age of 76 years (standard deviation 14 years). The most frequent achalasia subtype was type II (46%). Among 16 patients (37.2%) who underwent multiple sessions of EUS-BTID, the number of sessions ranged from 2 to 14, with the 14-session maximum occurring in a single patient. The average interval between repeat procedures across all patients was 273 days. The primary outcome of improved dysphagia scores, measured by the Eckardt score, was observed in 86.7% of patients, with 70.0% achieving scores indicative of complete remission. After EUS-BTID, the median Eckhardt score significantly improved from 6 (interquartile range, 4-7) to 2 (interquartile range, 1-4) (*P* < .0001). No serious adverse events were reported; only 2 patients (4.6%) experienced mild transient postprocedural symptoms, including chest discomfort and emesis.

**Conclusion:**

The novel EUS-BTID technique appears to be an effective and safe treatment option for achalasia. The combination of endoscopic ultrasound—guided Botox injection and balloon dilation offers significant improvements in symptom relief with a favorable safety profile and durability and can be applied in high-risk patients or probably as a bridge to more definitive therapy. Although our study provides encouraging evidence supporting the use of EUS-BTID, further research is required to validate these findings.

## Introduction

Achalasia is an esophageal motility disorder characterized by the inability of the lower esophageal sphincter (LES) to relax and the absence of normal organized esophageal peristalsis.^1^ This condition leads to progressive dysphagia, regurgitation, and significant discomfort, severely impacting quality of life.^2^ Achalasia is relatively rare, with a reported incidence of about 2 to 3 per 100,000 and a prevalence of 1 per 10,000.^3^ Current treatments aim to reduce LES pressure and facilitate esophageal emptying, which can be achieved by pharmacologic, endoscopic, or surgical means.^4^ Orally administered pharmacologic treatments such as calcium channel blockers or nitrates are no longer preferred because of the high incidence of side effects and their limited efficacy.^5^ Endoscopic methods are the current mainstay of achalasia management and include local administration of botulinum toxin (Botox; Allergan Aesthetics, Westport, Ireland) into the LES, pneumatic dilation (PD), and peroral endoscopic myotomy (POEM).^4^ Myotomy, whether performed by POEM or laparoscopic technique, remains the most effective long-term treatment for achalasia; however, these approaches are invasive, require significant expertise, and may not be appropriate for elderly or high-risk patients. PD and endoscopic local injection of Botox can offer a minimally invasive option in subjects who are unfit for surgery or where the expertise is unavailable.

Botox is a neurotoxic protein produced by the *Clostridium botulinum* and related species of bacteria that inhibit the release of acetylcholine from nerve endings.^4^ Botox injections work by temporarily paralyzing the LES muscle, thereby reducing its tone, and easing the passage of food and fluid.^6^ This method, although minimally invasive and effective, often requires repeat procedures because of its temporary nature.^6^ In addition, Botox injections traditionally have been performed by standard endoscopy, which lacks precision. Studies have shown that imprecise injections may result in submucosal or extramural delivery, potentially diminishing therapeutic efficacy. Kumar et al^6^ demonstrated that Botox injection into the LES induces targeted muscle paralysis, confirming that proper delivery to the muscularis propria is critical for its intended physiological effect and for avoiding unintended consequences such as hiatal paralysis or reflux. Pneumatic balloon dilation, in contrast, involves mechanically stretching the LES to disrupt the muscle fibers, providing more sustained relief but with potential risks such as perforation and postprocedural pain.^7^^,^^8^ PD involves stepwise use of large-diameter (30-40 mm), high-pressure balloons to disrupt LES fibers and carries risks such as perforation. By contrast, the controlled radial expansion (CRE) balloons used are through-the-scope devices with a maximum diameter of 20 mm that provide controlled radial expansion, representing a different safety and efficacy profile. Combining precise Botox injection and CRE balloon dilation could potentially enhance therapeutic outcomes by synergistically reducing LES pressure through both chemical and mechanical means. The development of endoscopic ultrasound (EUS) has expanded the capabilities of gastrointestinal endoscopy, allowing for more precise and targeted interventions.^9^ EUS-guided fine-needle injection (FNI) provides real-time imaging and improved accuracy in delivering therapeutic agents like Botox or chemotherapy directly into a target. EUS FNI can be a means to more precisely deliver Botox into the muscularis propria of the LES, thereby potentially improving efficacy.^10^ After Botox is injected into the muscularis propria of the LES, causing paralysis, subsequent dilation can result in a prolonged relaxation state. Coupling EUS FNI of Botox into the muscularis propria with balloon dilation just after the LES is paralyzed by Botox may offer a novel comprehensive approach to treating achalasia in patients who are at high risk for more invasive treatments.

This pilot study introduces a novel technique using EUS-guided FNI of Botox combined with CRE balloon dilation (EUS-BTID) for treating achalasia. We hypothesize that this combined approach will enhance the efficacy and safety profile compared with traditional methods of standard endoscopic Botox therapy for achalasia. This study aimed to assess the therapeutic effectiveness of EUS-BTID in terms of symptom relief, esophageal emptying, and LES pressure reduction. Secondary objectives included evaluating the safety profile and adverse events. By introducing and evaluating the EUS-BTID technique in this manner, this research aims to establish the safety and efficacy of this treatment modality for achalasia in high-risk patients with limitations for more definitive therapy.

## Methodology

This single-center retrospective study was conducted to evaluate the efficacy and safety of a novel technique using EUS-BTID for the treatment of achalasia and esophagogastric junction outflow obstruction. Patients were eligible for EUS-BTID if they had significant comorbidities limiting candidacy for definitive therapy (life expectancy ≤12 months, severe cardiopulmonary disease, or inability to hold anticoagulation for ≥48 hours), or if they declined more definitive therapies (POEM, Heller myotomy, or PD) after informed consent. Patients with manometric findings of esophagogastric junction outflow obstruction also were included if they presented with dysphagia and were treated with Botox and CRE balloon dilation. Additional inclusion criteria required an Eckardt score ≥5.

Exclusion criteria included previous esophageal surgery, other nonachalasia motility disorders, contraindications to Botox or balloon dilation, and inability to tolerate propofol anesthesia. The diagnosis of achalasia was confirmed by clinical presentation, high-resolution manometry, and radiographic findings.

The study protocol was approved by the Institutional Review Board at Parkview Health (Fort Wayne, Ind, USA). Data were collected through a review of electronic medical records. An electronic medical record search was performed using the terms “achalasia,” “balloon dilation,” “pneumatic dilation,” “botulinum toxin” or “Botox,” “endoscopic ultrasound,” and “EUS-BTID.” Demographic information, clinical characteristics, procedure details, and follow-up data were extracted.

The primary outcome was defined as improvement in dysphagia symptoms, measured by the Eckardt score (range 0-12), with a score ≤3 considered clinical remission. Adverse events were predefined and systematically recorded as part of outcome assessment. Secondary outcomes included adverse events.

Patients fasted overnight and were administered propofol anesthesia before the procedure. An initial esophagogastroduodenoscopy using a standard upper adult endoscope (Olympus GIF-180 or Olympus GIF-190; Olympus Corp, Center Valley, Pa, USA) was performed on all patients with careful attention to the squamocolumnar junction, top of gastric folds, and diaphragmatic hiatus. These landmarks were recorded along with any other routine upper-endoscopy findings. Biopsy samples were taken when clinically indicated. A linear array EUS scope (Olympus GF-UE190 or GF-UE180) was introduced into the esophagus. The gastroesophageal junction was identified, and a 22-gauge EUS fine-needle aspiration (Expect; Boston Scientific, Marlborough, Mass, USA) needle was used to inject 100 units of Botox type A into 4 quadrants of the LES under real-time EUS guidance (Figure 1, Figure 2, Figure 3).

**Figure 1:**
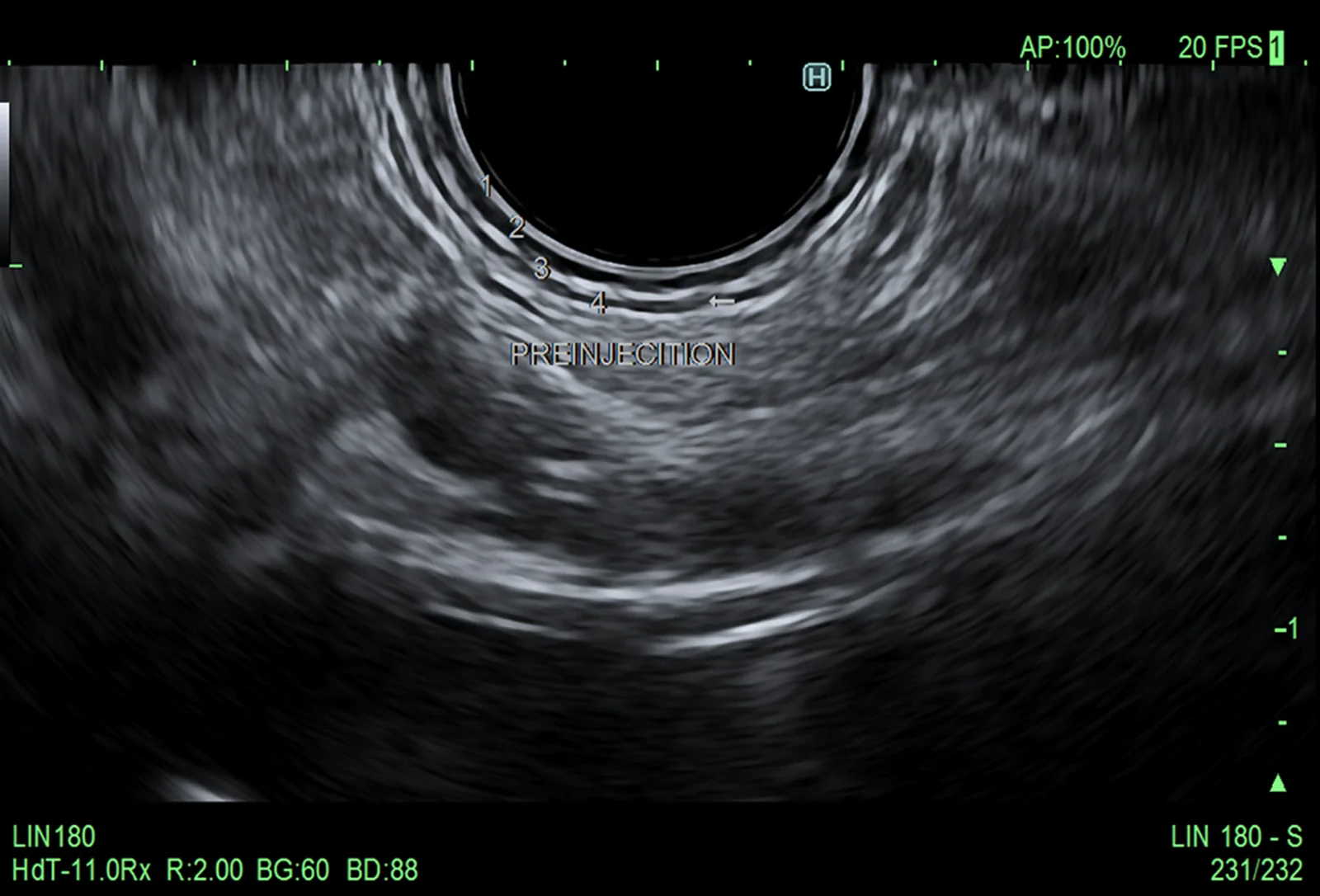
Endoscopic ultrasound image of the lower esophageal sphincter before botulinum toxin injection. Structures: interface (**1**), mucosa (**2**), submucosa (**3**), circular muscle layer (muscularis propria) (**4**). *Arrow* = circular muscle layer, the target for botulinum toxin injection.

**Figure 2:**
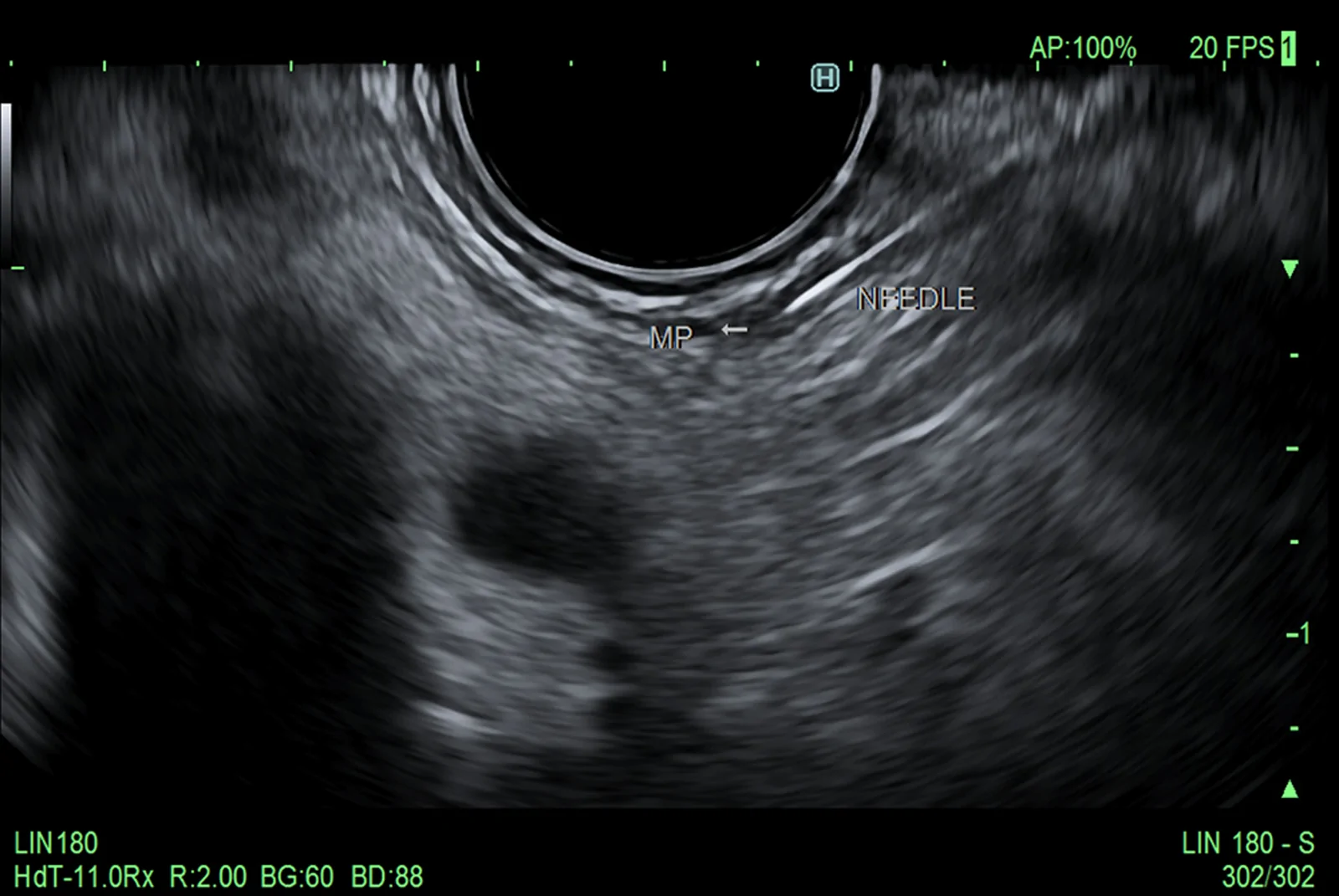
Endoscopic ultrasound image of the lower esophageal sphincter after botulinum toxin injection. The *arrow* indicates the muscularis propria (MP) layer of the esophagus. The echogenic needle (NEEDLE) is visualized entering the muscle layer under direct endoscopic ultrasound guidance (*white arrow*), ensuring accurate targeting during endoscopic ultrasound—guided fine-needle injection of botulinum toxin combined with controlled radial expansion balloon dilation therapy.

**Figure 3:**
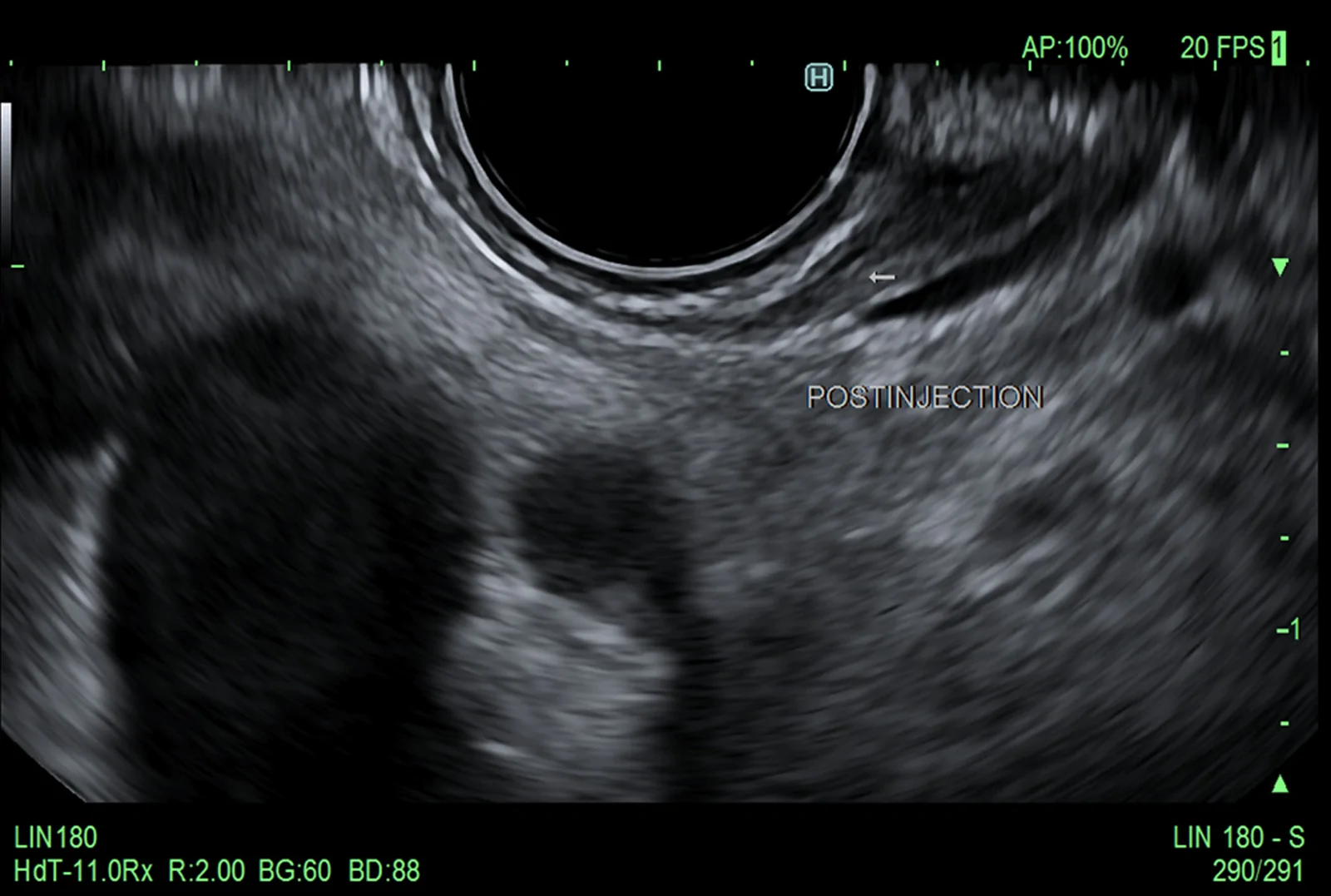
Endoscopic ultrasound image of the lower esophageal. *Arrow* points to muscularis propria after botulinum toxin injection.

After the Botox injection, a catheter-based balloon dilator was inserted through the channel of a standard adult upper endoscope (Olympus GIF-180 or Olympus GIF-190) to the gastroesophageal junction. The balloon was inflated to a diameter of 20 mm and maintained for 1 minute to achieve adequate dilation. Patients were observed in the recovery unit for standard of care (30 minutes and once fully recovered from a routine endoscopy standpoint) postprocedure. Vital signs and symptoms were monitored, and patients were instructed to follow a soft diet for 24 hours, gradually advancing to a regular diet. Patients were followed at 1 month, 3 months, 6 months, and 12 months postprocedure. Follow-up assessments included clinical evaluation, dysphagia scoring using the Eckardt score, esophageal manometry to assess LES pressure and esophageal peristalsis, and endoscopic examination to evaluate mucosal integrity and any potential adverse events. Adverse events were recorded and classified according to the American Society for Gastrointestinal Endoscopy lexicon.^11^ Adverse events were categorized as immediate (within 24 hours) or delayed (after 24 hours) and graded by severity.

Data were analyzed using the Statistical Package for the Social Sciences, Version 27 (IBM Corp; Armonk, NY, USA) and Stata version 18 (StataCorp LLC, College Station, Tex, USA). Quantitative variables were expressed as mean (standard deviation [SD]) or median (interquartile range [IQR]), whereas qualitative variables were presented as frequencies and percentages. The Shapiro-Wilk test was used to assess normality. Wilcoxon signed-rank tests were used to compare change in Eckardt scores, with *P* < .05 considered statistically significant. Bonferroni correction was used for multiple comparisons. Although this was a retrospective study and data collection was not prospectively limited by sample size, a post hoc power analysis was conducted to assess the adequacy of the sample. A sample size of 25 patients was determined to provide 80% power (α = 0.05) to detect a moderate effect size (Cohen’s d = 0.6) using a 2-tailed Wilcoxon signed-rank test.

## Results

Our data were based on a total of 43 patients, with a mean age of 76 years (SD: 14 years at the time of first procedure performance). A total of 23 (53.5%) patients were female, and the female-to-male ratio was 1.15 to 1. Nine (20.9%) patients were deceased at the time of data analysis, with all deaths occurring as a result of natural causes and unrelated to the procedure itself. The mean age at the time of death was 82 years, with 8 (88.9%) patients among the deceased being male. With regard to subtype, type II achalasia was the most frequently encountered disorder, accounting for 20 (46.5%) cases (Table 1).

**Table 1:** Patient and procedure characteristics

Variable	Value
Age, years, mean (SD)	76 (14)
Sex, n (%)	
Female	23 (53.5%)
Male	20 (46.5%)
Type of achalasia, n (%)	
Type I	3 (7%)
Type II	20 (46%)
Type III	6 (14%)
EGJOO with achalasia variant	14 (33%)
Preprocedural Eckardt score for 70% (n = 30) patients involved in the study∗, median (IQR)	6 (4, 7)
Postprocedural Eckardt score for 70% of the patients involved in the study, median (IQR)	2 (1, 4)
Number of procedures, n (%)	
1	27 (62%)
2	13 (30%)
3	2 (5%)
14	1 (2%)
Average time between procedures, mean (SD)	273 (171) days
Immediate adverse events (<24 hours), n (%)	
Pain	1 (2.3%)
Emesis	1 (2.3%)
Minimal improvement in dysphagia symptoms	2 (5%)
Delayed adverse events (>24 hours), n (%)	None reported

*EGJOO*, Esophagogastric junction outflow obstruction; *IQR*, interquartile range; *SD*, standard deviation.

∗ Follow-up Eckardt scores were available for 70% of patients; missing data were due to loss to follow-up or incomplete documentation.

A total of 16 (37.2%) patients underwent more than 1 session of EUS-BTID, with a minimum of 2 sessions and a maximum of 14 sessions that were performed on 1 patient. One patient with type III achalasia underwent 14 EUS-BTID sessions over 5 years as a result of recurrent symptoms and lack of surgical candidacy. Each session yielded transient symptom improvement, and repeat treatment was pursued on the basis of patient preference and clinical need. The mean time between consecutive procedures was 273 days (SD, 171 days). The postprocedural Eckardt score was assessed at a median of 3 months after EUS-BTID (range, 1-6 months). Data for Eckardt score were only available for 30 (69.8%) patients. Of these, 26 (86.7%) patients had some degree of improvement, of whom 21 (70.0%) had improvements in Eckardt score ≤3, which was suggestive of complete remission of achalasia. Unfortunately, 2 (6.7%) patients had worsening symptoms, whereas 2 (6.7%) had no change. After EUS-BTID, the median Eckardt score significantly improved from 6 (IQR, 4-7) to 2 (IQR, 1-4) (*P* < .0001). Similar findings were observed in subgroup analyses by patients who underwent 1 session of EUS-BTID versus more than 1 session of EUS-BTID. Among patients who underwent 1 session (n = 18), median Eckardt score significantly improved from 6 (IQR, 4-7) to 2 (IQR, 1-5) (*P* = .0006). Among patients who underwent more than 1 session (n = 12), median Eckardt score significantly improved from 5 (IQR, 4.5-6) to 2 (IQR, 1-3.5) (*P* = .006).

EUS-BTID was safe; there were no serious adverse events. Mild adverse events were reported in 2 patients (4.6%), including 1 case of transient postprocedural chest discomfort and 1 case of postprocedural emesis, both of which resolved without intervention within 24 hours. No delayed or 30-day adverse events were identified.

## Discussion

The results of our study demonstrate that the novel technique of EUS-BTID is both effective and safe for the treatment of achalasia. The majority of patients experienced significant symptom relief, with substantial improvements in dysphagia scores reported. Among patients who required repeat EUS-BTID, the average time between procedures was 273 days. This interval provides an approximate reflection of clinical response durability in that subgroup. Repeat procedures were performed on the basis of documented symptom recurrence during follow-up. This aligns with our hypothesis that the combined approach would provide enhanced therapeutic benefits over traditional methods of subcutaneous injection with standard endoscopy. EUS-BTID would have potential applications in suboptimal candidates for POEM, Heller myotomy, and PD.

Improvement in dysphagia scores was observed in 86.7% of patients for whom follow-up Eckardt data were available, with 70.0% achieving scores indicative of complete remission. Eckardt scores were significantly reduced among patients after EUS-BTID from a median preprocedural score of 6 to a postprocedural score of 2. These findings suggest that EUS-BTID provides effective relief of achalasia symptoms, potentially because of the synergistic effects of Botox and mechanical dilation. Botox reduces LES tone by chemically paralyzing the muscle, whereas balloon dilation disrupts muscle fibers, allowing for more sustained relief. The efficacy of this combination is further supported by the mean duration between consecutive procedures (273 days), indicating a prolonged period of symptom control.

The conventional Botox technique involves using a standard endoscope and injection needle to inject Botox into the area presumed to be the LES. This technique lacks precision, and the major shortcoming of this technique is the limited durability of symptom relief lasting between 1 and 6 months.^4^ In a Cochrane Database of Systematic Review that compared conventional endoscopic Botox injection with PD, the authors observed similar rates of clinical success at 4 weeks but a reduction after that in clinical success with Botox at 6 months (80.70% vs 51.80%) and 12 months (73.30% vs 37.50%).^12^ This same study also showed that Botox injection was safe with no reported adverse events, while there were 3 cases of perforation among the 188 patients who underwent PD.^12^

In patients with elevated risk for POEM or Heller myotomy, or in locations where those techniques are not available, PD is the preferred option. PD causes disruption of the LES fibers through intraluminal dilation of a pressurized balloon. The conventional approach is stepwise dilations starting at 30 mm (sizes 30-, 35-, and 40-mm diameter) sequentially until the desired clinical response is achieved.^13^ Guidelines recommend PD as an initial management strategy for achalasia, with laparoscopic Heller myotomy and POEM preferred where the expertise is available.^14^ PD, however, carries a risk of approximately 2.8% for perforation and 2% for clinically significant bleeding. EUS-BTID does not involve using a pneumatic balloon; it uses a through-the-scope CRE balloon with a maximal dilating diameter of 20 mm. This lesser degree of LES dilation in EUS-BTID could potentially reduce the risk of serious adverse effects compared with PD. The combined use of Botox injection and CRE balloon dilation may offer enhanced symptom relief by targeting both neural and mechanical contributors to LES dysfunction. Although standard endoscopic Botox injection is generally considered safe, EUS-guided delivery may offer improved precision in targeting the muscularis propria, potentially enhancing efficacy. In our experience, the safety profile of the EUS-BTID technique was favorable, with only a minority of patients experiencing transient postprocedural chest pain. No serious adverse events, such as perforation or severe postprocedural pain requiring intervention, were reported. This safety outcome is better than more invasive methods such as POEM, Heller myotomy, or PD. EUS-guided injection techniques have previously been described for other gastrointestinal motility disorders, including gastroparesis, where direct myenteric delivery of Botox under EUS guidance was shown to be feasible and potentially beneficial. These studies support the plausibility and safety of EUS-guided toxin injection in motility-related conditions.^15^

Compared with traditional endoscopic Botox injection, EUS guidance may improve precision of delivery into the muscularis propria, whereas the addition of balloon dilation provides mechanical disruption of the lower esophageal sphincter. This combined approach may therefore offer a safe and effective alternative in high-risk patients or serve as a bridge to definitive therapy, although more prospective studies are needed to validate these findings.

Despite these promising results, our study has a few limitations. First, its retrospective design and single-center setting may limit the generalizability of the findings. The sample size was modest, and follow-up data were incomplete for a subset of patients, particularly with regard to postprocedural Eckardt scores and manometric outcomes. In addition, variability in the number and timing of EUS-BTID sessions introduces potential confounding when interpreting treatment durability and efficacy. Although some patients underwent repeat procedures on the basis of symptom recurrence, standardized follow-up intervals were not uniformly applied. Quality-of-life metrics were not consistently captured, and postprocedural manometry was not available in most cases. This study represents an early evaluation of a novel technique. Future prospective, multicenter studies with standardized symptoms and motility assessments are warranted to validate these findings and further define the role of EUS-BTID in the management of achalasia.

## Conclusions

The novel EUS-BTID offers a promising therapeutic option for achalasia, particularly in high-risk patients or as a bridge to more definitive therapy. Although our early experience suggests this approach is feasible and safe, future prospective multicenter studies with standardized follow-up are needed to validate long-term outcomes and clarify its role within the therapeutic algorithm for achalasia.

## Patient consent

The study was approved by the Parkview Health Institutional Review Board. The requirement for individual informed consent for participation was waived by the Institutional Review Board because of the retrospective nature of the study and the use of existing, deidentified clinical data. Written informed consent for the endoscopic procedure was obtained from all patients before the procedure.

## Disclosure

All authors disclosed no financial relationships.
